# Supplementary material for: Ancient DNA reveals diverse community organizations in the 5th millennium BCE Carpathian Basin
Source: Nat Commun. 2025 Jun 24;16:5318. doi: 10.1038/s41467-025-60368-2 (PMC12187924; doi:10.1038/s41467-025-60368-2)
Supplement: Supplementary file 2 — Description of Additional Supplementary Files [file 41467_2025_60368_MOESM2_ESM.docx]

File Name: Supplementary Data 1
Description: Laboratory and bioinformatic summary.

File Name: Supplementary Data 2
Description: Archaeological summary.

File Name: Supplementary Data 3
Description: PCA outlier detection and test for homogeneity of the studied groups.

File Name: Supplementary Data 4
Description: *f*4-statistics.

File Name: Supplementary Data 5
Description: Summary of the qpAdm tests.

File Name: Supplementary Data 6
Description: hapROH and IBD *N_e_* estimation summary.

File Name: Supplementary Data 7
Description: Nodes of the IBD network, corresponding to Fig. 4 and Supplementary Fig. 6.

File Name: Supplementary Data 8
Description: IBD statistics.

File Name: Supplementary Data 9
Description: List of IBD cliques, counted based on the graph shown in Fig. 4A.

File Name: Supplementary Data 10
Description: Summary of the uniparental haplogroups and their statistics.

File Name: Supplementary Data 11
Description: IBD connections over 2x12 cM, with minimum 100 cM total IBD sharing

File Name: Supplementary Data 12
Description: Details of the genetic relatedness analyses.

File Name: Supplementary Data 13

Description: Oligonucleotides and reagents used in the ancient DNA workflow.
