# Supplementary material for: Ancient DNA reveals diverse community organizations in the 5th millennium BCE Carpathian Basin
Source: Nat Commun. 2025 Jun 24;16:5318. doi: 10.1038/s41467-025-60368-2 (PMC12187924; doi:10.1038/s41467-025-60368-2)
Supplement: Supplementary file 16 — Reporting Summary [file 41467_2025_60368_MOESM16_ESM.pdf]

nature portfolio

Corresponding author(s): Anna Szécsényi-Nagy, David Reich, Zsuzsanna Siklósi

Last updated by author(s): 04-04-2025

Reporting Summary

Nature Portfolio wishes to improve the reproducibility of the work that we publish. This form provides structure for consistency and transparency in reporting. For further information on Nature Portfolio policies, see our Editorial Policies and the Editorial Policy Checklist.

Statistics

For all statistical analyses, confirm that the following items are present in the figure legend, table legend, main text, or Methods section.

n/a	Confirmed	
[ ]	[x]	The exact sample size (n) for each experimental group/condition, given as a discrete number and unit of measurement
[ ]	[x]	A statement on whether measurements were taken from distinct samples or whether the same sample was measured repeatedly
[ ]	[x]	The statistical test(s) used AND whether they are one- or two-sided<br>*Only common tests should be described solely by name; describe more complex techniques in the Methods section.*
[x]	[ ]	A description of all covariates tested
[x]	[ ]	A description of any assumptions or corrections, such as tests of normality and adjustment for multiple comparisons
[ ]	[x]	A full description of the statistical parameters including central tendency (e.g. means) or other basic estimates (e.g. regression coefficient) AND variation (e.g. standard deviation) or associated estimates of uncertainty (e.g. confidence intervals)
[ ]	[x]	For null hypothesis testing, the test statistic (e.g. F, t, r) with confidence intervals, effect sizes, degrees of freedom and P value noted<br>*Give P values as exact values whenever suitable.*
[ ]	[x]	For Bayesian analysis, information on the choice of priors and Markov chain Monte Carlo settings
[x]	[ ]	For hierarchical and complex designs, identification of the appropriate level for tests and full reporting of outcomes
[x]	[ ]	Estimates of effect sizes (e.g. Cohen's d, Pearson's r), indicating how they were calculated

Our web collection on statistics for biologists contains articles on many of the points above.

Software and code

Policy information about availability of computer code

Data collection	For data collection, we did not use software but a publicly available data base (AADR) and further literature data, publicly available sequence data. We selected ancient reference data based on chronology and geography.
Data analysis	Data were analyzed by open source softwares, all listed in the Methods section of the paper. We used OxCal 4.4.4. for radiocarbon dating. Raw sequence data were analysed as written in https://github.com/dReichLab/ADNA-Tools and https://github.com/dReichLab/adna-workflow. Furthermore, we used ANGSD (v.0.939-10-g21ed01c, htslib 1.14-9-ge769401) hapCon (hapROH package v.0.60), TTNe (v0.0.1), samtools, contamMix, Ugene (v40), DnaSP (v5), PopArt (v1.7), AdmixTools (v.7.0.1), GLIMPSE (v.1.1.1), bcftools mpileup (v1.10.2), KIN (3.1.1), Python 3.7, 3.12 packages pandas (1.3.5 and 2.2.2), numpy (1.21.6), scipy (1.7.3 and 1.13.1). We visualized results using networkx (2.3), matplotlib (3.5.3 and 3.9.2), seaborn (0.9.0 and 0.13.2). We used KIN (v3.1.1) READ (v1.01), ancIBD (v0.5), Gephi (v0.10.1) and hapROH (v0.60), HaploGrep (v.2.1.25) Yleaf (v.2.2) softwares. They are all referenced with version numbers in the Methods. No novel software tool was developed for this paper, just short scripts were created for basic statistical calculations and plotting. These are deposited at at GitHub (github.com/ArchGenIn/Szecsenyi-Nagy_2025) and at Zenodo (DOI: 10.5281 /zenodo.15221967).

For manuscripts utilizing custom algorithms or software that are central to the research but not yet described in published literature, software must be made available to editors and reviewers. We strongly encourage code deposition in a community repository (e.g. GitHub). See the Nature Portfolio guidelines for submitting code & software for further information.

Data

Policy information about availability of data

All manuscripts must include a data availability statement. This statement should provide the following information, where applicable:

- Accession codes, unique identifiers, or web links for publicly available datasets
- A description of any restrictions on data availability
- For clinical datasets or third party data, please ensure that the statement adheres to our policy

All data needed to evaluate the results of the paper are present in the paper and/or the Supplementary Materials. Ancient genome sequences were uploaded to ENA (European Nucleotide Archive), under the accession number of PRJEB86386 (https://www.ebi.ac.uk/ena/browser/view/PRJEB86386). The AADR v54.1 dataset is publicly available at https://dataverse.harvard.edu/dataset.xhtml?persistentId=doi:10.7910/DVN/FFIDCW. 1000 Genome Project data as the reference panel for the imputation was taken from https://www.internationalgenome.org/data-portal/data-collection/30x-grch38.
Data required to generate all figures in the manuscript are available in Supplementary Data files and Source Data files. Open science principles require making all data used to support the conclusions of a study maximally available, and we support these principles here by making fully publicly available not only the digital copies of molecules (the uploaded sequences) but also the molecular copies (the ancient DNA libraries themselves, which constitute molecular data storage). Those researchers who wish to carry out deeper sequencing of libraries published in this study should make a request to corresponding author, D.R. We commit to granting requests as long as the libraries remain preserved in our laboratories, with no requirement that we be included as collaborators or co-authors on any resulting publications.

Research involving human participants, their data, or biological material

Policy information about studies with human participants or human data. See also policy information about sex, gender (identity/presentation), and sexual orientation and race, ethnicity and racism.

Reporting on sex and gender	We analysed ancient individuals from the prehistoric era. We defined sex of these individuals using anthropological methods and DNA testing. We compared biological sex with archaeological objects (grave goods) that might signalize gender identity, but this was not a key part of our paper.
Reporting on race, ethnicity, or other socially relevant groupings	We cross-checked archaeological categories with genetic results, but did not use the archaeological classes or pottery styles as groups in the analyses, but treated individual data independently. We used geography and chronology of the burials as relevant grouping factors.
Population characteristics	The groups we analyzed consist of both males and females, approximately in equal proportions. As we did not study diseases or health conditions, other covariates are not relevant to this study.
Recruitment	We aimed at a representative sampling for DNA analyses form certain cemeteries. Our selection was not influenced by any preconception, but rather aimed at covering different levels and periods of the cemeteries. We selected graves to represent both female and male, and burials with wealthy and modest grave-goods and different parts of the burial grounds.
Ethics oversight	We followed the ethical standards of aDNA research introduced by Alpaslan-Roodenberg et al. 2021 in Nature. We received formal permission for analysis of all samples from local authorities (Satu Mare County Museum, Budapest History Museum, Hungarian Natural History Museum, and Intercisa Museum).

Note that full information on the approval of the study protocol must also be provided in the manuscript.

Field-specific reporting

Please select the one below that is the best fit for your research. If you are not sure, read the appropriate sections before making your selection.

☒ Life sciences ☐ Behavioural & social sciences ☐ Ecological, evolutionary & environmental sciences

For a reference copy of the document with all sections, see nature.com/documents/nr-reporting-summary-flat.pdf

Life sciences study design

All studies must disclose on these points even when the disclosure is negative.

Sample size	We did not perform a priori sample size calculations. Our aim was to achieve equal sampling across the sites: Aszód, Csőszhalom, and Basatanya, with 30-30 samples from each (due to budgetary reasons, we have not sampled complete sites). We sampled all available and excavated graves from Urziceni.
Data exclusions	We excluded certain samples based on the laboratory screening, and some data from the analyses based on data quality, contamination estimates, and the prerequisites of specific statistical tests. All exclusions and criteria are detailed in the Methods section.
Replication	We performed NGS, generating millions of sequencing reads, that approach includes replicates in itself. The resulting dataset is extensive, with the report providing essential quality and sequence coverage information required for assessment. Statistical methods (f-statistics) used are widely applied and published.

Randomization	We did not randomize the samples, as it is not applicable in our case, but treated them in most of the analyses individually. Possible covariates were considered, and they did not influence the results.
Blinding	We conducted the laboratory analyses with blinding, using lab codes and co-analyzing samples alongside extraction blanks. Bioinformatic processing was also performed using lab codes, ensuring it was not influenced by a priori expectations. Other forms of blinding were not necessary for our analyses.

Reporting for specific materials, systems and methods

We require information from authors about some types of materials, experimental systems and methods used in many studies. Here, indicate whether each material, system or method listed is relevant to your study. If you are not sure if a list item applies to your research, read the appropriate section before selecting a response.

Materials & experimental systems

n/a	Involved in the study
☒	☐ Antibodies
☒	☐ Eukaryotic cell lines
☐	☒ Palaeontology and archaeology
☒	☐ Animals and other organisms
☒	☐ Clinical data
☒	☐ Dual use research of concern
☒	☐ Plants

Methods

n/a	Involved in the study
☒	☐ ChIP-seq
☒	☐ Flow cytometry
☒	☐ MRI-based neuroimaging

Palaeontology and Archaeology

Specimen provenance	Anthropological materials of the following sites are stored in the collection of Department of Anthropology, Hungarian Natural History Museum, Hungarian National Museum Public Collection Centre: Aszód-Papi földek, Polgár-Csőszhalom, Polgár-Nagykasziba, Rákóczifalva-Bagi föld, site 8, Tiszapolgár-Basatanya.<br>Anthropological material of Budapest-Albertfalva-Hunyadi János út site is stored in the prehistoric collection of the Budapest History Museum, Aquincum Museum in Budapest, Hungary.<br>Anthropological material of Iváncsa-Lapos is stored in Intercisa Museum, Dunaújváros, Hungary<br>Anthropological material of Urziceni-Vama site is sored in the collection of the Satu Mare County Museum in Romania.
Specimen deposition	Not the specimens, but the DNA libraries are deposited for free access, as written in the Ethical Declaration part of the paper. The samples may be accessed in the museums by their skeletal code and grave number listed in Supplementary Data 1-2.
Dating methods	Human remains were radiocarbon dated, samples deriving from the petrous bones or ribs. Radiocarbon measurements were performed in the Poznan Radiocarbon Laboratory, the Pennsylvania State University Radiocarbon Laboratory, VERA Laboratory Universität Wien, HEKAL Laboratory in Debrecen. Radiocarbon data were calibrated with using the IntCal20 curve and the Oxcal v4.4.4. software.

☒ Tick this box to confirm that the raw and calibrated dates are available in the paper or in Supplementary Information.

Ethics oversight	The individuals studied in this research were analyzed with the goal of minimizing damage, with permission obtained from local authorities in each respective location. Each sample is represented by stewards, such as archaeologists or museum curators, who are either listed as Authors or acknowledged in the Acknowledgments section. No ethical commission oversaw the research as a whole, as it was not required.

Note that full information on the approval of the study protocol must also be provided in the manuscript.

Plants

Seed stocks	*Report on the source of all seed stocks or other plant material used. If applicable, state the seed stock centre and catalogue number. If plant specimens were collected from the field, describe the collection location, date and sampling procedures.*
Novel plant genotypes	*Describe the methods by which all novel plant genotypes were produced. This includes those generated by transgenic approaches, gene editing, chemical/radiation-based mutagenesis and hybridization. For transgenic lines, describe the transformation method, the number of independent lines analyzed and the generation upon which experiments were performed. For gene-edited lines, describe the editor used, the endogenous sequence targeted for editing, the targeting guide RNA sequence (if applicable) and how the editor was applied.*
Authentication	*Describe any authentication procedures for each seed stock used or novel genotype generated. Describe any experiments used to assess the effect of a mutation and, where applicable, how potential secondary effects (e.g. second site T-DNA insertions, mosiacism, off-target gene editing) were examined.*
